# Social Connectedness and Cognitive Resilience: The Role of Social Cognition

**DOI:** 10.1093/geroni/igaf122.906

**Published:** 2025-12-31

**Authors:** Anne Krendl, Lucas Hamilton, Brea Perry

**Affiliations:** Indiana University Bloomington, Bloomington, Indiana, United States; Augustana University, Sioux Falls, South Dakota, United States; Indiana University Bloomington, Bloomington, Indiana, United States

## Abstract

Social connectedness is a modifiable lifestyle factor that may also delay the progression of Alzheimer’s disease. However, the mechanism by which this occurs is not well-understood. Our work examines whether older adults’ social cognitive function is the mechanism by which social connectedness confers resilience against cognitive decline. We focus specifically on theory of mind – the ability to infer what others are thinking and feeling – because it is a core social cognitive skill that is impaired in healthy aging. We examined whether theory of mind mediated the relationship between older adults’ social connectedness and cognition using data collected from 305 community-dwelling older adults. 110 of those participants completed follow-up social network interviews and cognitive assessments about 1.5 years later to determine whether baseline social connectedness and theory of mind predicted cognitive change. Results revealed that theory of mind accounted for 32% of the relationship between social connectedness and overall cognition, even when covarying age, gender, education, and a control task. The effects were particularly robust for episodic memory and language. Longitudinal analyses replicated this pattern. An additional 55 other older adults completed an experimental manipulation in which participants were asked to describe a task to a close and distant network member. We found that older adults with larger, less dense social networks provided more details to distant versus very close network members. Together, these results suggest that theory of mind may provide the mechanism through which social connectedness confers cognitive resilience.

